# Power Allocation Scheme for Non-Orthogonal Multiple Access in Underwater Acoustic Communications

**DOI:** 10.3390/s17112465

**Published:** 2017-10-27

**Authors:** Jinyong Cheon, Ho-Shin Cho

**Affiliations:** School of Electronics Engineering, Kyungpook National University, Daegu 41566, Korea; jycheon@ee.knu.ac.kr

**Keywords:** power allocation, non-orthogonal multiple access (NOMA), underwater acoustic sensor networks (UWASNs)

## Abstract

In this paper, we propose a power allocation scheme for non-orthogonal multiple access (NOMA) in underwater acoustic sensor networks (UWASNs). The existing terrestrial sum-rate maximization (SRM) power allocation scheme suffers from the degradation of the overall sum-rate in UWASNs due to wasteful resource created by unequal transmission times between each transmission path. To address this issue, we propose the equal transmission times (ETT) power allocation scheme, which can prevent wasteful resource generation by guaranteeing equal transmission times between each transmission path. ETT considers the number of packets waiting for transmission in the sender’s buffer for creating equal transmission times. Numerical results show that the proposed ETT outperforms SRM in terms of the overall sum-rate, while having nearly identical maximum sum-rate to the SRMs.

## 1. Introduction

Among the envisioned medium access control (MAC) protocols for the fifth generation of mobile communication, the non-orthogonal multiple access (NOMA) protocol is one of the most promising candidate techniques, as it has been shown to increase cell capacity. To support multiple users, the NOMA protocol allocates two-dimensional time-frequency resources that are superposed and transmitted to multiple users whose channel gain differences are very large. Each receiver node can decode an individual packet from packets that are superposed in the power domain, by using the method of successive interference cancellation (SIC). Consequently, the NOMA protocol can achieve high cell capacity. On the other hand, conventional orthogonal multiple access (OMA) protocols allocate resources to each user either in time or in frequency or in code, which limits the cell capacity.

In underwater environments, acoustic waves are used for communications instead of radio frequency (RF) waves due to the absorption and diffusion of RF waves. Additionally, underwater ambient noise is not white noise, i.e., it has frequency-dependent characteristics. Thus, underwater acoustic channels suffer from low propagation speed, narrow frequency bandwidth, and low data rate. These unique channel conditions necessitate the modification of conventional terrestrial MAC protocols before they are applied to underwater acoustic sensor networks (UWASNs).

The paper examines the feasibility of the NOMA protocol in an underwater channel that has distance/frequency-dependent attenuation and frequency-dependent ambient noise. Additionally, the performance degradation issues found in existing sum-rate maximization (SRM) power allocation schemes for downlink underwater NOMA are addressed. Wasteful resources caused by unequal transmission times between NOMA-paired channels may have an adverse effect on the data rate of multiplexed channels. Hence, this paper proposes a new scheme named equal transmission times (ETT) power allocation to eliminate wasteful resources. To guarantee equal transmission times, ETT first calculates the data rate required for each path, based on the number of packets waiting in the sender’s buffer. Subsequently, ETT calculates the transmission power required to achieve the calculated data rate. Equal transmission times are guaranteed, and the proposed ETT can prevent generation of wasteful resources. In terms of the overall sum-rate, which is defined as the mean of the sum of the data rates for two paired channels during a total transmission time, we observe that ETT outperforms existing SRM scheme irrespective of the number of packets for each path in the sender’s buffer.

This paper is organized as follows. First, we provide an overview on the characteristics of the underwater channel and on the clustered UWASNs in Section 2. Section 3 gives a brief overview of problems with the existing SRM in underwater environments. The details of the proposed ETT are described in Section 4. Numerical results are presented in Section 5. Finally, conclusions are drawn in Section 6.

## 2. An Overview of the Characteristics of the Underwater Channel and the Clustered UWASNs

### 2.1. The Characteristics of the Underwater Channel

In this paper, we consider Thorp’s underwater channel model [1], which is based on empirical data. Thorp’s model provides the attenuation and the ambient noise for the underwater channel. The attenuation of an acoustic wave is influenced by the frequency and the communication distance between the sender node and the receiver node. Hence, the overall attenuation can be expressed as a function of the distance (l) and the frequency (f), which is given by Equation (1),

(1) $$A\left(l,f\right)=A_{0}l^{k}a{\left(f\right)}^{\frac{l}{10^{3}}}$$

where l is a distance (m) between the sender and the receiver node, f is signal frequency (kHz), $A_{0}$ is the normalizing constant, and k is the spreading factor. The spreading factor has a value between 1 and 2 depending on the depth. k=1 means a cylindrical spreading which characterizes shallow water communications. k=2 means a spherical spreading which characterizes deep water communications. Generally, k=1.5 is often considered as practical spreading. We therefore assume the spreading factor as 1.5 and the normalizing constant as 30 dB. a(f) is the absorption coefficient and is expressed in decibels per kilometer,

(2) $$10\log a\left(f\right)=0.11\frac{f^{2}}{1+f^{2}}+44\frac{f^{2}}{4100+f^{2}}+2.75\times 10^{-4}f^{2}+0.003.$$

For lower than a few hundred Hz, the following formula may be used:

(3) $$10\log a\left(f\right)=0.002+0.11\frac{f^{2}}{1+f^{2}}+0.011f^{2}.$$

**Figure 1:**
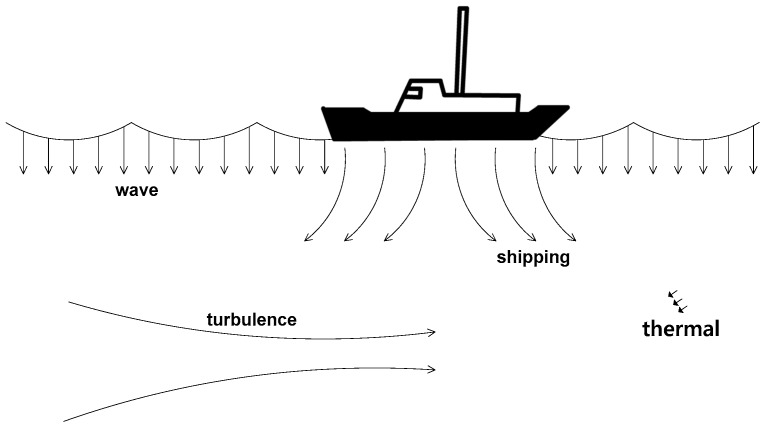
The ambient noise sources: turbulence, shipping, wave, and thermal.

Figure 1 illustrates the ambient noise sources. There are four ambient noise sources in the ocean: turbulence, shipping, waves, and thermal noise of the molecules. Equation (4) gives the power spectrum density (PSD) of these noise sources in dB re μ Pa^2^/Hz [2],

(4) $$\{\begin{array}{l} 10\log N_{t}\left(f\right)=17-30\log f\\ 10\log N_{s}\left(f\right)=40+20\left(s-0.5\right)+26\log f-60\log\left(f+0.03\right)\\ 10\log N_{w}\left(f\right)=50+7.5w^{\frac{1}{2}}+20\log f-40\log\left(f+0.4\right)\\ 10\log N_{th}\left(f\right)=-15+20\log f \end{array}.$$

where $N_{t}\left(f\right)$, $N_{s}\left(f\right)$, $N_{w}\left(f\right)$, and $N_{th}\left(f\right)$ denote the noise components of turbulence, shipping, waves, and thermal noise, respectively. The PSD of total ambient noise $N_{total}\left(f\right)$, which is sum of the PSDs of four sources, can be described by

(5) $$N_{total}\left(f\right)=N_{t}\left(f\right)+N_{s}\left(f\right)+N_{w}\left(f\right)+N_{th}\left(f\right).$$

**Figure 2:**
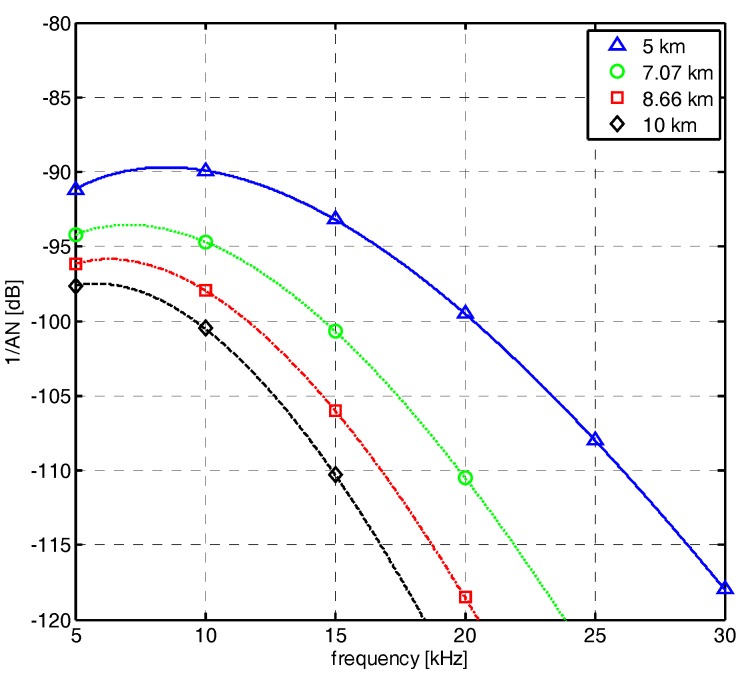
AN product according to distance and frequency.

The ambient noise decays with a frequency that is limiting the system bandwidth. By considering the attenuation A(l,f) and the ambient noise $N_{total}\left(f\right)$, the signal to noise ratio (SNR) can be calculated by

(6) $$SNR\left(l,f\right)=\frac{P_{sender}}{A\left(l,f\right)N_{total}\left(f\right)}$$

where $P_{sender}$ is a PSD of the transmitted signal by the sender node. The frequency dependent part of the signal to noise ratio (SNR), $A\left(l,f\right)N_{total}\left(f\right)$, is called the AN product. Figure 2 shows the AN product according to communication distance and frequency.

### 2.2. Clustered Underwater Acoustic Sensor Networks (UWASNs)

**Figure 3:**
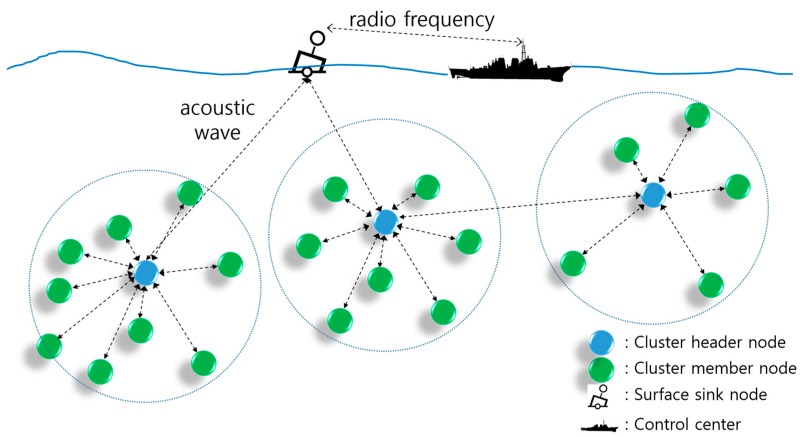
The clustered underwater acoustic sensor network (UWASN) considered in this paper.

In UWASNs, the energy efficiency is widely considered as the most important challenge. To prolong the network lifetime, various approaches have been proposed to solve this issue in clustered networks [3,4,5]. Figure 3 shows the clustered UWASN considered in this paper. The clustered UWASN comprises a surface sink node and the underwater sensor nodes. Every sensor node has the same capabilities such as storage, processing power, communication range, and battery life. Each sensor node can become a cluster header (CH) node or a cluster member (CM) node, depending on the situation. The CM nodes gather event or environmental information and forward this data to its CH node. i.e., as a coordinator node of the cluster, the CH node aggregates data from the CM nodes in their cluster or sometimes transmits data to its CM nodes for controlling the cluster. Periodically, each CH node transmits aggregated data to the surface sink node through single-hop or multi-hop transmission. Finally, the surface sink node transfers aggregated data to the control center using RF waves.

The intra-cluster and inter-cluster communications are different from the communication between the surface sink node and the control center. Because of the half-duplex property and space-time uncertainty [6] of the underwater channel, a variety of MAC protocols are proposed for UWASNs to prevent collisions. The underwater MAC protocols can be classified into three broad types [7]: contention-free MAC protocol, contention-based MAC protocol, and hybrid MAC protocol. However, most of these OMA protocols are operated in a manner that the resource is temporarily unavailable to other nodes except communicating nodes. Thus, if a NOMA protocol is applied to UWASNs by exploiting the attenuation and noise characteristics of the underwater channel which is mentioned above, multiple nodes can share the same two-dimensional time-frequency resource according to the channel gain difference.

The sharing of resources can enhance the performance of underwater communications. The performance of NOMA highly depends on node pairing and power allocation schemes [8]. Thus, when we find the power allocation ratio for paired nodes, calculating the received power and the expected data rate at each paired node is essential. However, conventional power allocation schemes for the terrestrial NOMA are not suitable for the underwater channel which has low data traffic. Therefore, in this paper, we propose a novel power allocation scheme that can be used for downlink NOMA for clustered UWASNs. This scheme assumes that the cluster header has already chosen the paired cluster member nodes. In other words, the node pairing scheme does not fall within the scope of this paper.

## 3. SRM Power Allocation Scheme in Underwater Channels and Their Issues

The conventional OMA protocols have some weakness when they are applied in the underwater channel. Time division multiple access (TDMA) needs accurate timing synchronization and a guard time to avoid collisions. These degrade the performance of the TDMA because of low channel utilization. Frequency division multiple access (FDMA), which use frequency bands exclusively at the same time, is not suitable for the narrow bandwidth underwater channel. In the case of code division multiple access (CDMA), the near-far problem is one of the major issues. In the near-far problem, the receiver cannot detect the weak signal in the presence of a strong signal. To overcome this issue, the sender node requires an extra overhead for the power control technique to ensure equal received power. In contrast, NOMA exploits the gain difference between the receiver nodes. This characteristic is suitable for the underwater channel that has a severe attenuation.

**Figure 4:**
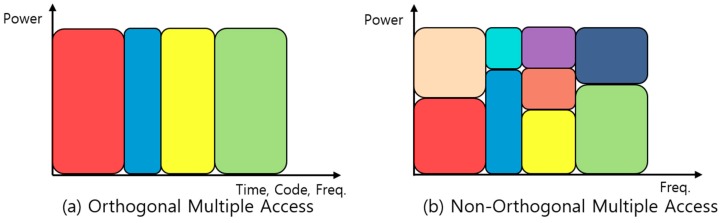
The difference between orthogonal multiple access (OMA) and non-orthogonal multiple access (NOMA), the resource allocated to different user is colored differently: (**a**) Orthogonal Multiple Access; (**b**) Non-Orthogonal Multiple Access.

Regarding resource allocation, there is a significant difference between the OMA and NOMA protocol as shown in Figure 4 where the resource allocated to different user is colored differently. OMA protocols in Figure 4a exclusively allocate resources such as time, frequency, and code to each user. Theoretically, there is no interference among users so a receiver can simply detect each user’s packet. Hence because of exclusive resource allocation, the maximum number of users being supported is limited. In contrast, as illustrated in Figure 4b, the NOMA protocol allocates same two-dimensional time-frequency resource to multiple users in the power domain. The NOMA protocol allocates the same resource exploiting the characteristics of channel difference, which allows multiple users who have a significant channel gain difference to share the same resource. Therefore the number of users being supported is not strictly limited. Unlike the OMA protocol, the NOMA protocol needs an elaborate user signal detection process named SIC at the receiver node side. Through the SIC, each user can decode an individual packet from the superposed packets in the power domain and therefore a high cell capacity is achieved.

**Figure 5:**
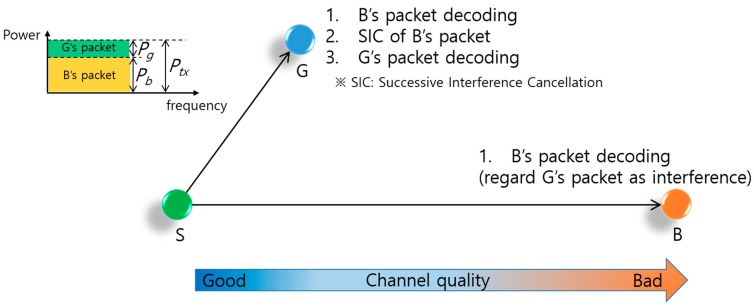
Two receiver nodes downlink NOMA.

Figure 5 provides an example of the downlink NOMA transmission via power domain multiplexing between a sender node S and two receiver nodes G and B. Among the two receiver nodes, G has good channel quality as it is located near to S, and B has bad channel quality because of severe attenuation commensurate with its distance from S. In general, node S pairs two receiver nodes that have large differences in their channel gains and then transmits data packets to the paired nodes concurrently at the same frequency. To achieve collision-free concurrent transmissions, the paired nodes’ packets are superposed in the power domain, wherein S allocates a larger transmission power ($P_{b}$) to the bad quality channel transmission and a smaller transmission power ($P_{g}$) to the good quality channel transmission without exceeding the transmission power constraint ($P_{tx}$), as shown in the upper-left part of Figure 5. There is a direct association between the power allocation ratio ($\gamma =P_{g}/P_{tx}$) and the sum-rate, which is defined by $R_{g}+R_{b}$, where $R_{g}$ and $R_{b}$ are the data transmission rates of the good and bad quality channels, respectively. Therefore, finding the optimized γ in terms of the sum-rate is critical for NOMA performance. Superposed packets are decoded in the following manner. Node B decodes its packet by considering G’s packet as interference. On the other hand, node G has to decode B’s packet first. Subtracting the decoded packet from the superposed packets, node G can then decode its packet from the subtracted packet, which is the summation of its packet and the ambient noise. This procedure is called SIC [9].

Most studies on power allocation for NOMA have focused on maximizing the sum-rate. In [10] the authors proposed two sub-optimal power allocation schemes based on the users’ instantaneous channel state information (CSI) in a sub-carrier based NOMA system. In [11] the authors first studied the ergodic capacity maximization problem for the Rayleigh fading multiple-input multiple-output (MIMO) NOMA systems. In this literature, the authors proposed both optimal and low complexity sub-optimal power allocation schemes to maximize the ergodic capacity under the conditions with a total transmit power constraint and minimum rate constraint of the weak user. The sum rate maximization of a multiple-input single-output (MISO) downlink NOMA system has been studied in [12]. The authors used a minorization-maximization algorithm to solve the downlink sum-rate maximization problem. In [13] the authors proposed a sub-optimal water filling based power allocation scheme to improve the total achieved system throughput. This scheme is operated in two stages: the water filling–based inter sub-band power allocation and the adaptive intra sub-band power allocation. The formulation of an optimization problem for maximizing the sum capacity has been studied in [14] for the single-input single-output (SISO) method. This paper considered maximizing the sum capacity under a total power constraint and a quality of service (QoS) condition of each user.

However, since SRM is based on the assumption that paired transmissions last until the end of transmissions, the maximum sum-rate cannot be sustained in the case of unequal transmission times between the paired channels when one of the paired channel completes the transmission before the other. Hence, resource waste occurs while only one channel is in transmission. Resource waste means the resource is available but cannot be used by any other nodes (including the sender node). Especially in UWASNs, resource waste may severely degrade the performance due to the low data rate and long propagation delay.

**Figure 6:**
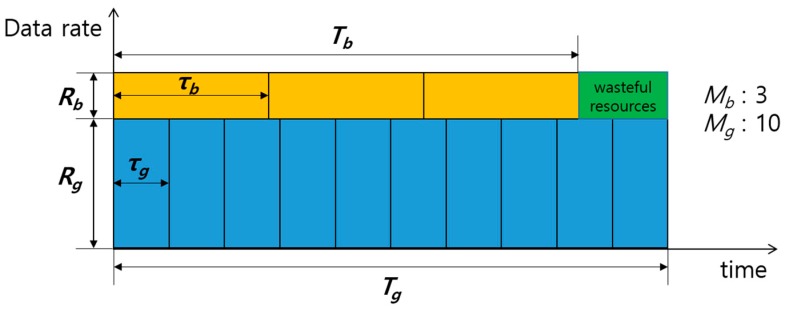
Wasteful resources occurring due to unequal transmission times.

Figure 6 shows an example where resource waste is generated due to unequal transmission times. For simplicity, the packet size in number of bits, $L_{p}$, is considered to be constant. Packet transmission times of nodes G and B, which are denoted by $\tau_{g}$ and $\tau_{b}$, respectively, are obtained by

(7) $$\begin{array}{l} \tau_{g}=\frac{L_{p}}{R_{g}}\\ \tau_{b}=\frac{L_{p}}{R_{b}} \end{array}.$$

**Figure 7:**
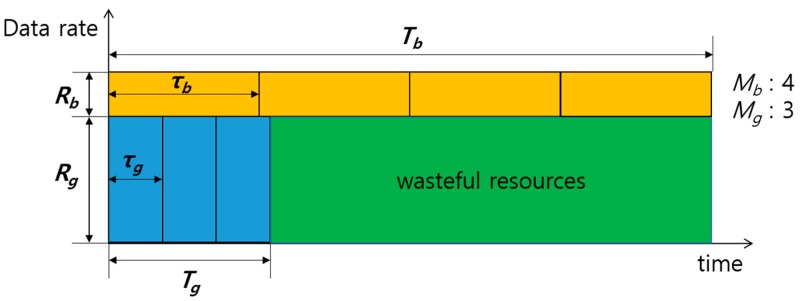
Wasteful resources occurring in sum-rate maximization (SRM) when $M_{g}$ and $M_{b}$ are small.

Thus, the total transmission times of G and B, which are denoted by $T_{g}$ and $T_{b}$, respectively, are obtained by

(8) $$\begin{array}{l} T_{g}=\tau_{g}\times M_{g}\\ T_{b}=\tau_{b}\times M_{b} \end{array},$$

where $M_{g}$ and $M_{b}$ are the number of packets transmitted to nodes G and B in the current transmission, respectively. The discrepancy in transmission times, $\delta_{T}\left(=\left|T_{g}-T_{b}\right|\right)$, causes the aforementioned resource waste. In this case, even though one of the transmissions is complete, to avoid a collision the other nodes cannot use resources for transmitting until the sender nodes’ total transmission time ($\max\left(T_{g},T_{b}\right)$) elapse. In general, the sum-rate is maximized when $R_{g}$ is much greater than $R_{b}$ (i.e., $\tau_{g}<<\tau_{b}$) [15]. Even under such conditions, if the number of packets destined for G and B in the buffer of node S is very large, we can easily make $\delta_{T}$ small enough to be negligible by adjusting $M_{g}$ and $M_{b}$. However, in UWASNs where the traffic generation is infrequent, the number of packets is likely to be small, and thus $\delta_{T}$ could become large, as shown in Figure 7, where $M_{g}=3$, $M_{b}=4$, and $\tau_{g}<<\tau_{b}$. 

Since SRM allocates transmission power without considering $M_{g}$ and $M_{b}$, the discrepancy $\delta_{T}$ is unavoidable, and sometimes can be very large. In our proposed ETT, we determine the transmission power that makes $T_{g}$ and $T_{b}$ equal, depending on $M_{g}$ and $M_{b}$, to eliminate wasteful resources.

## 4. Proposed ETT Power Allocation Scheme

**Figure 8:**
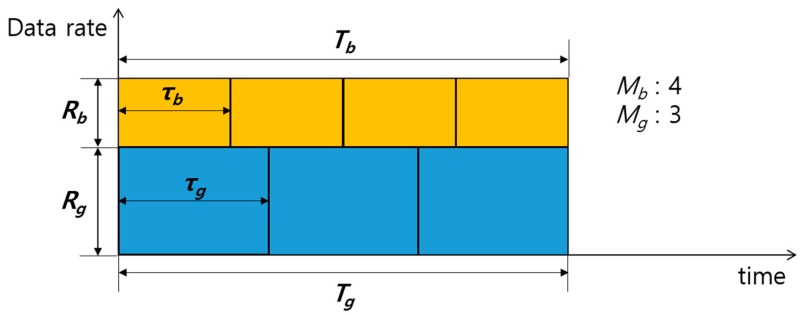
The time occupancy of nodes G and B in the proposed equal transmission times (ETT).

Figure 8 presents the equal transmission times of nodes G and B in the proposed ETT under the same condition as Figure 7. To make $\delta_{T}$ equal to 0 (i.e., $T_{g}=T_{b}$), the transmission power allocation ratio (γ) should be determined to satisfy the following relationship:

(9) $$\frac{R_{g}}{R_{b}}=\frac{M_{g}}{M_{b}}.$$

However, the γ obtained from Equation (9) should be located within an operating range of γ, which guarantees the minimum SNR at which both the nodes, G and B, are capable of decoding the received signal. Thus, we first find the operating range and then choose within the operating range an appropriate γ associated with Equation (9).

**Figure 9:**
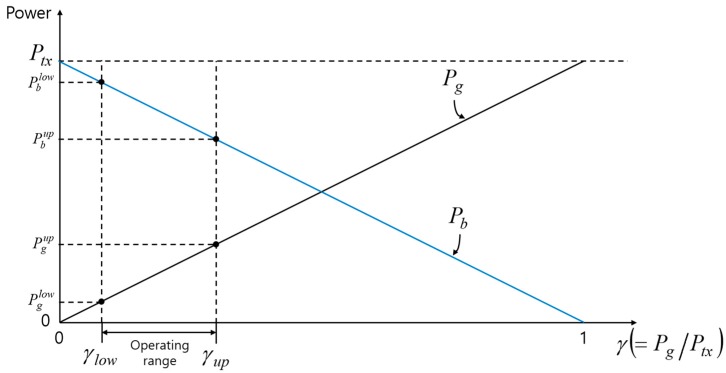
The relationship between power allocation and operating range.

Figure 9 illustrates the relationship between power allocation and operating range. The lower bound and the upper bound of the operating range are denoted by $\gamma_{low}$ and $\gamma_{up}$, respectively. $\gamma_{low}$ is the point where the SNR of node G ($SNR_{g}$) becomes the minimum SNR. At the point $\gamma_{low}$, the transmission powers toward nodes G and B are denoted by $P_{g}^{low}$ and $P_{b}^{low}\left(=P_{tx}-P_{g}^{low}\right)$, respectively. Similarly, $\gamma_{up}$ is the point where the SNR of node B ($SNR_{b}$) becomes the minimum SNR. The corresponding transmission powers to nodes G and B are $P_{g}^{up}$ and $P_{b}^{up}$, respectively. In the following paragraphs, we show how by $P_{g}^{low}$ and $P_{b}^{low}$ can be determined.

The SNR of node G at $\gamma_{low}$ is obtained by

(10) $$SNR_{g}^{low}=\frac{\int_{B}S_{g}^{low}\left(f\right)A^{-1}\left(l_{g},f\right)df}{\int_{B}N\left(f\right)df},$$

where B is the frequency bandwidth, $S_{g}^{low}\left(f\right)$ is the PSD of the transmitted signal toward G, A(l,f) is the attenuation with respect to the distance l and the frequency f, $l_{g}$ is the distance between S and G, and N(f) is the PSD of the ambient noise. The PSD of transmission power follows the water-filling principle [16] given by

(11) S(f)+A(l,f)N(f)=k,

where k is a constant depending on the allocated transmission power. Using Equation (11), Equation (10) can be rewritten as

(12) $$\begin{array}{cc} SNR_{g}^{low} & =\frac{\int_{B}\left[k-A\left(l_{g},f\right)N\left(f\right)\right]A^{-1}\left(l_{g},f\right)df}{\int_{B}N\left(f\right)df}\\ & =k\frac{\int_{B}A^{-1}\left(l_{g},f\right)df}{\int_{B}N\left(f\right)df}-1 \end{array}.$$

Therefore, from Equation (12), *k* is obtained by

(13) $$\begin{array}{cc} k & =\frac{\int_{B}N\left(f\right)df}{\int_{B}A^{-1}\left(l_{g},f\right)df}\times \left(SNR_{g}^{low}+1\right)\\ & \equiv k_{g}^{low} \end{array},$$

where k is newly denoted by $k_{g}^{low}$. Then, the transmission power $P_{g}^{low}$ that corresponds to the minimum SNR at node G is given by

(14) $$P_{g}^{low}=\int_{B}S_{g}^{low}\left(f\right)df=k_{g}^{low}\times B-\int_{B}A\left(l_{g},f\right)N\left(f\right)df.$$

Consequently, the transmission power $P_{b}^{low}$ at $\gamma_{low}$ is obtained by

(15) $$P_{b}^{low}=P_{tx}-P_{g}^{low}.$$

As a next step, we find the data rates associated with $\gamma_{low}$. According to the Shannon capacity [17], the data rate $R_{g}$ and $R_{b}$ at $\gamma_{low}$ are given by

(16) $$R_{g}^{low}=\int_{B}\log_{2}\left[1+\frac{S_{g}^{low}\left(f\right)/A\left(l_{g},f\right)}{N\left(f\right)}\right]df,$$

(17) $$R_{b}^{low}=\int_{B}\log_{2}\left[1+\frac{S_{b}^{low}\left(f\right)/A\left(l_{b},f\right)}{S_{g}^{low}/A\left(l_{b},f\right)+N\left(f\right)}\right]df,$$

where $S_{b}^{low}\left(f\right)$ is the PSD of the transmitted signal to node B at $\gamma_{low}$, and $l_{b}$ is the distance between S and B. Similar to the case of $\gamma_{low}$, we find the corresponding power and the data rate at $\gamma_{up}$. First, the SNR of node B at $\gamma_{up}$ is obtained by

(18) $$SNR_{b}^{up}\left(dB\right)=\frac{\int_{B}S_{b}^{up}\left(f\right)A^{-1}\left(l_{b},f\right)df}{\int_{B}S_{g}^{up}\left(f\right)A^{-1}\left(l_{b},f\right)+N\left(f\right)df}.$$

Unlike the case of the $\gamma_{low}$, the denominator of Equation (18) is the overall noise at B, which is the sum of the attenuated $P_{g}^{up}$ and the ambient noise. If we substitute the denominator of Equation (18) with $\int_{B}N_{overall}\left(l_{g},l_{b},f\right)df$, Equation (18) can be rewritten as

(19) $$\begin{array}{cc} SNR_{b}^{up}\left(dB\right) & =\frac{\int_{B}S_{b}^{up}\left(f\right)A^{-1}\left(l_{b},f\right)df}{\int_{B}N_{overall}\left(l_{g},l_{b},f\right)df}\\ & =k_{b}^{up}\frac{\int_{B}A^{-1}\left(l_{b},f\right)df}{\int_{B}N_{overall}\left(l_{g},l_{b},f\right)df}-1 \end{array},$$

where $k_{b}^{up}$ is the parameter k of Equation (13) for the case of $\gamma_{up}$. Then, $k_{b}^{up}$ is obtained as

(20) $$\begin{array}{cc} k_{b}^{up} & =\frac{P_{b}^{up}+\int_{B}A\left(l_{b},f\right)N_{overall}\left(l_{g},l_{b},f\right)df}{B}\\ & =\frac{P_{tx}+\int_{B}A\left(l_{b},f\right)N\left(f\right)df}{B} \end{array}.$$

Thus, the $P_{b}^{up}$ and the $P_{g}^{up}$ are obtained by

(21) $$P_{b}^{up}=\int_{B}S_{b}^{up}\left(f\right)df=k_{b}^{up}\times B-\int_{B}A\left(l_{b},f\right)N_{overall}\left(l_{g},l_{b},f\right)df,$$

(22) $$P_{g}^{up}=P_{tx}-P_{b}^{up}.$$

The data rates $R_{g}^{up}$ and $R_{b}^{up}$ are obtained in the same way as Equations (16) and (17).

**Figure 10:**
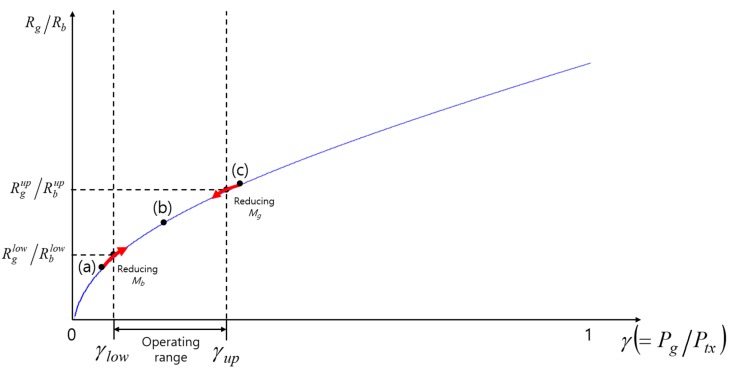
The change of the data rates ratio with varying γ.

Figure 10 shows the change of the data rates ratio ($R_{g}/R_{b}$) with varying γ. If the ratio, $R_{g}/R_{b}$, obtained from Equation (9) when $M_{g}$ and $M_{b}$ are chosen to carry all the packets waiting in the buffers destined for nodes G and B, respectively, falls within the operating range of γ (case (b)), we only have to choose the corresponding transmit power. On the other hand, if the ratio $R_{g}/R_{b}$ falls outside the operating range (left-hand side of $\gamma_{low}$ (case (a)) or right-hand side of $\gamma_{up}$ (case (c))), we keep reducing $M_{b}$ for case (a) or $M_{g}$ for case (c) by 1 until the ratio $R_{g}/R_{b}$ falls within the operating range.

## 5. Numerical Results

In this section, we analyze the performance of ETT compared to SRM. The parameters we used are summarized in Table 1.

**Figure 11:**
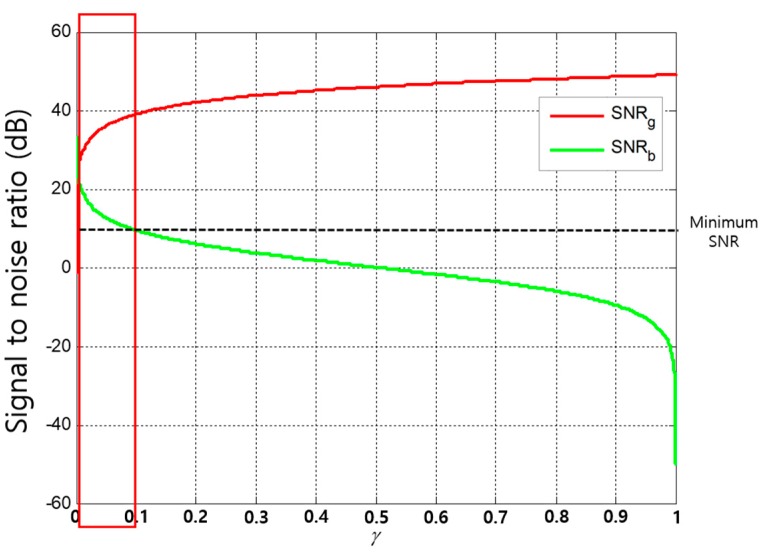
The operating range of NOMA protocol in UWASNs.

Figure 11 shows the variation of each receiver node’s SNR with varying γ. In this analysis, for the minimum SNR of 10 dB, we observe that the operating range of γ for the underwater NOMA to be a very narrow range (rectangular outline in Figure 11), unlike the terrestrial NOMA, because of the severe attenuation related to the communication distance and the frequency, which are prevalent issues in the underwater channel.

**Figure 12:**
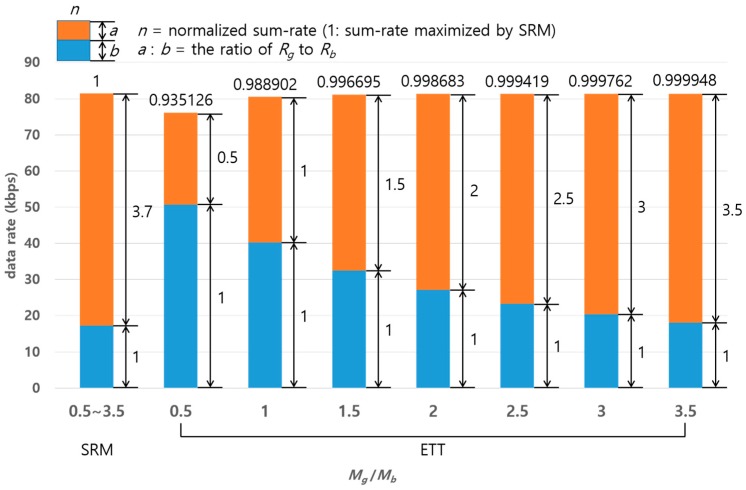
A comparison in terms of sum-rate with varying the ratio of packets for paired two channels.

Figure 12 shows a comparison of SRM and ETT in terms of sum-rate varying the packet ratio ($M_{g}/M_{b}$). The numbers on the top of the bars indicate the sum-rate normalized by the maximum sum-rate of SRM. Meanwhile, the numbers on the side of the bars indicate the ratio of data rate of $R_{g}$(upper) and $R_{b}$(lower). The numbers on the botton of the bar indicates the packet ratio $M_{g}/M_{b}$. It is shown that the normalized sum-rate of ETT is approximately equivalent to that of SRM even supporting various data rate ratios ($R_{g}/R_{b}$) led by the packet ratio ($M_{g}/M_{b}$). On the other hand, SRM can achieve the maximum sum-rate under a specific condition of data rate which is 3.7:1 in this example regardless of $M_{g}/M_{b}$. Thus, if the packet ratio does not meet the data rate ratio 3.7:1, SRM’s sum-rate cannot be fully utilized. Note that the reason why ETT has comparatively low sum-rate at 0.5 of $M_{g}/M_{b}$ in this analysis is that NOMA inherently shows a better performance when a higher power is allocated to a good quality channel.

Naturally, the transmission times $T_{g}$ and $T_{b}$ are different. Thus, from the point of view of this paper, the overall sum-rate is defined by

(23) $$R_{overall}=\frac{\tau_{g}R_{g}M_{g}+\tau_{b}R_{b}M_{b}}{\max\left(T_{g},T_{b}\right)}.$$

**Figure 13:**
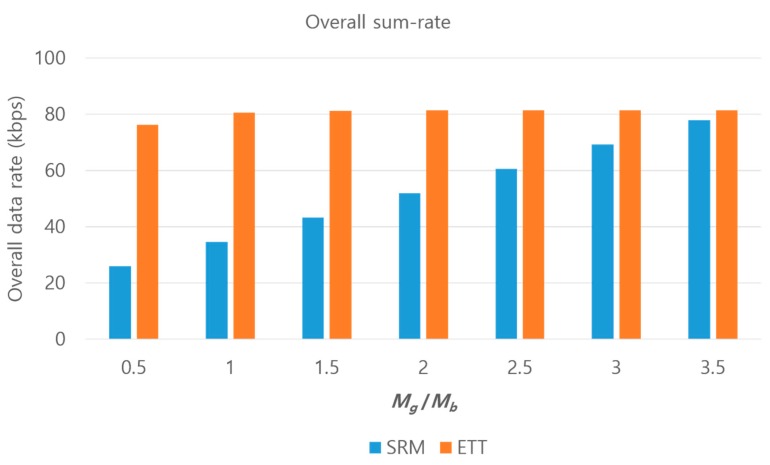
A comparison in terms of overall sum-rate with varying the ratio of packets for each path.

Figure 13 shows the comparison in terms of the overall sum-rate. We observed that ETT outperforms SRM; especially when $M_{g}/M_{b}$ is small, the performance difference becomes larger because the transmission times discrepancy, $\delta_{T}$, becomes larger under the condition of $\tau_{g}<<\tau_{b}$. ETT finds the appropriate γ, depending on the number of packets, as well as guaranteeing equal transmission times and thus mitigates the occurrence of wasteful resources.

## 6. Conclusions

In this paper, we proposed an ETT power allocation scheme for resolving the overall sum-rate degradation issues related to the SRM power allocation scheme in UWASNs. Unlike SRM, the proposed ETT finds an appropriate transmission power for each paired channel to guarantee equal transmission times considering the number of packets to each transmission path. As a result, ETT can eliminate wasteful resources through equal transmission times. Numerical analysis shows that while the sum-rate is analogous to that of SRM, the overall sum-rate of ETT is much larger than that of SRM for varying packet ratio scenarios.

Most studies on MAC protocols for UWASNs have focused on how to apply OMA protocols to the underwater channel. Thus, these studies have exclusively provided the solution for assigning and using limited resources. However, we believe that the NOMA protocol that allows a simultaneous sharing of an identical resource according to the channel gain difference can present an advanced solution for UWASNs which experience a severe attenuation according to distance. The performance of NOMA is determined by node pairing and power allocation. Our proposed power allocation scheme will be helpful in solving the issues of overall sum-rate degradation for the low traffic underwater channel.

In this paper, we dealt with the situation of two receiver nodes downlink NOMA. However, with the same concept of ETT, *N* receiver nodes downlink NOMA will be able to operate without significant complexity. To further our research, we intend to extend ETT to general *N* receiver nodes downlink NOMA.

## Figures and Tables

**Table 1:** Parameters for numerical analysis.

Parameters	Unit	Value
Bandwidth (*B*)	kHz	10–15
Distance between S and G ($l_{g}$)	m	100
Distance between S and B ($l_{b}$)	m	900
Transmission power ($P_{tx}$)	Watts	2
Minimum SNR	dB	10
Data packet size ($L_{p}$)	bits	8000
